# Differences in Mitochondrial Membrane Potential Identify Distinct Populations of Human Cardiac Mesenchymal Progenitor Cells

**DOI:** 10.3390/ijms21207467

**Published:** 2020-10-10

**Authors:** Elisa Gambini, Ilenia Martinelli, Ilaria Stadiotti, Maria Cristina Vinci, Alessandro Scopece, Luana Eramo, Elena Sommariva, Jessica Resta, Sabrina Benaouadi, Elisa Cogliati, Adolfo Paolin, Angelo Parini, Giulio Pompilio, Frederique Savagner

**Affiliations:** 1 Vascular Biology and Regenerative Medicine Unit, Centro Cardiologico Monzino-IRCCS, Via Carlo Parea 4, 20138 Milan, Italy; ilaria.stadiotti@ccfm.it (I.S.); cristina.vinci@ccfm.it (M.C.V.); alessandro.scopece@ccfm.it (A.S.); luanaeramo.le@gmail.com (L.E.); elena.sommariva@ccfm.it (E.S.); jessica.resta@inserm.fr (J.R.); giulio.pompilio@ccfm.it (G.P.); 2 Institute of Metabolic and Cardiovascular Diseases (I2MC), Institut National de la Santé et de la Recherche Médicale (INSERM), Université de Toulouse, 31432 Toulouse, France; ilenia.martinelli@inserm.fr (I.M.); sabrina.benaouadi@inserm.fr (S.B.); angelo.parini@inserm.fr (A.P.); frederique.savagner@inserm.fr (F.S.); 3 Treviso Tissue Bank Foundation, Via Antonio Scarpa 9, 31100 Treviso, Italy; ecogliati@fbtv-treviso.org (E.C.); adolfo.paolin54@gmail.com (A.P.); 4 Dipartimento di Scienze Cliniche e di Comunità, Università degli Studi di Milano, Via Festa del Perdono 7, 20122 Milan, Italy

**Keywords:** cardiac mesenchymal progenitor cells, metabolism, mitochondrial membrane potential, TMRM, cell fate, differentiation

## Abstract

Adult human cardiac mesenchymal progenitor cells (hCmPC) are multipotent resident populations involved in cardiac homeostasis and heart repair. Even if the mechanisms have not yet been fully elucidated, the stem cell differentiation is guided by the mitochondrial metabolism; however, mitochondrial approaches to identify hCmPC with enhanced stemness and/or differentiation capability for cellular therapy are not established. Here we demonstrated that hCmPCs sorted for low and high mitochondrial membrane potential (using a lipophilic cationic dye tetramethylrhodamine methyl ester, TMRM), presented differences in energy metabolism from preferential glycolysis to oxidative rates. TMRM-high cells are highly efficient in terms of oxygen consumption rate, basal and maximal respiration, and spare respiratory capacity compared to TMRM-low cells. TMRM-high cells showed characteristics of pre-committed cells and were associated with higher in vitro differentiation capacity through endothelial, cardiac-like, and, to a lesser extent, adipogenic and chondro/osteogenic cell lineage, when compared with TMRM-low cells. Conversely, TMRM-low showed higher self-renewal potential. To conclude, we identified two hCmPC populations with different metabolic profile, stemness maturity, and differentiation potential. Our findings suggest that metabolic sorting can isolate cells with higher regenerative capacity and/or long-term survival. This metabolism-based strategy to select cells may be broadly applicable to therapies.

## 1. Introduction

Adult stem cells are widespread in all tissues of the human body where they exist in a quiescent state. Once activated by injury mediators or disease, they promote tissue repair and regeneration contributing to restoration of normal tissue function [1]. Among adult stem cells, mesenchymal stem cells (MSCs) comprise a heterogeneous population of multipotent adult mesodermal progenitors whose numerous biological properties, such as low immunogenic and high immunosuppressive properties [2], broad differentiation potential, both in vitro and in vivo [3,4,5,6], and easy isolation [7,8], make them attractive candidates for cell-based therapies. Human cardiac mesenchymal progenitor cells (hCmPCs) are a novel MSC population arising from cardiac tissue [9]. Compared to bone marrow (BM)-derived MSCs, these cells are more prone to express cardiovascular markers and differentiate into cardiomyocytes, while exhibiting a reduced ability to differentiate along the chondrogenic and osteogenic lineages [9,10]. For this latest characteristic, hCmPCs have been used for cell-based therapy in regenerative medicine trials, e.g., CADUCEUS (NCT00893360), CAREMI (NCT02439398), and ALLSTAR (NCT01458405), where their safety and regenerative potential was clearly assessed. Despite numerous in vivo studies evidenced by low engraftment ability, poor survival and almost the complete differentiation failure of MSCs [11,12,13], suggesting the secretion of biologically active molecules as mode of action, there is also some notable evidence of direct contribution of MSCs to tissue regeneration [14,15]. Such discrepancy could rely on different cell delivery, e.g., systemic vs. local, as reviewed in [16], able to promote one function over the other. To this regard, the factors able to affect hCmPC engraftment optimization and differentiation can be grouped into patient factors and cell factors, such as culturing conditions and cell purity [17]. Indeed, although uniformly positive for mesenchymal markers, hCmPCs could represent a blend cell population endowed of different stemness maturity and differentiation potential. To this regard, recent studies have underlined the key role of mitochondrial biogenesis in stem cell differentiation [18,19], showing a strong coupling between intrinsic metabolic parameters and stem cell fate [20]. Consistently, stem cells differentiation is characterized by oxidative phosphorylation (OXPHOS) upregulation and glycolysis downregulation [21]. Indeed, low mitochondria number with reduced inner mitochondrial membrane potential (Δψm) and oxidative capacity [21], can explain for glycolytic metabolism of stem cells. Noteworthy, Δψm is now predictive of stem cell self-renewal and lineage commitment, providing a useful guide to select optimal stem cell population with enhanced stemness and/or differentiation capability for cellular therapy [22]. To date, very few studies were aimed at investigating the metabolism of resident hCmPCs [23,24,25]. Here, for the first time, by TMRM, a fluorescent dye previously used to sort stem cells based on their Δψm [19,22,26], we identified two hCmPC populations characterized by different metabolic profile, stemness maturity and differentiation potential. Our findings suggest that metabolic sorting can complement sorting based on conventional cell surface markers in identifying cells with higher regenerative capacity and/or long-term survival with broader applicability in a variety of clinical settings.

## 2. Results

### 2.1. Surface Marker Expression

**Figure 1:**
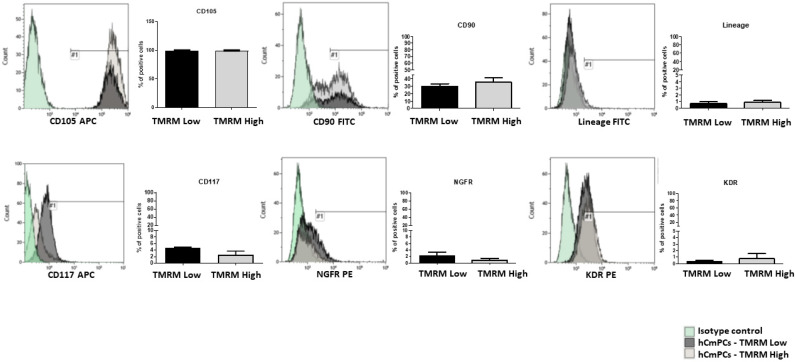
Characterization of tetramethylrhodamine methyl ester (TMRM)-low and TMRM-high cell populations by fluorescent activated cell sorting (FACS) analysis. Bar graphs shows the expression of the mesenchymal markers CD105, CD90 and Lineage (CD2, CD3, CD11b, CD14, CD15, CD16, CD19, CD56, CD123, and CD235a), the stem cells markers CD117 and nerve growth factor receptor (NGFR) and the endothelial marker vascular endothelial growth factor receptor 2 (VEGFR-2)/kinase insert domain receptor (KDR) in human cardiac mesenchymal progenitor cells (hCmPCs) sorted by TMRM-low and high expression. Data are represented as mean ± SD. *n* = 3 per group.

To establish if different metabolic states prime hCmPCs to different fate choices, we stained hCmPCs with TMRM, a mitochondrial membrane potential sensitive dye, and sorted cell populations with intrinsically different levels of mitochondrial oxygen consumption. To do this, we loaded hCmPCs with TMRM and separated cells using a fluorescent activated cell sorting (FACS) for either high or low fluorescence (see Supplementary Figure S1 for sorting strategy). Immediately after sorting, the gated TMRM-high and TMRM-low populations were analyzed by FACS to assess differences in the expression of mesenchymal, stem cell, and endothelial markers. The results showed that these two populations were phenotypically indistinguishable (Figure 1) except for a slightly higher CD117 expression in TMRM-low population.

### 2.2. Energy Metabolism

**Figure 2:**
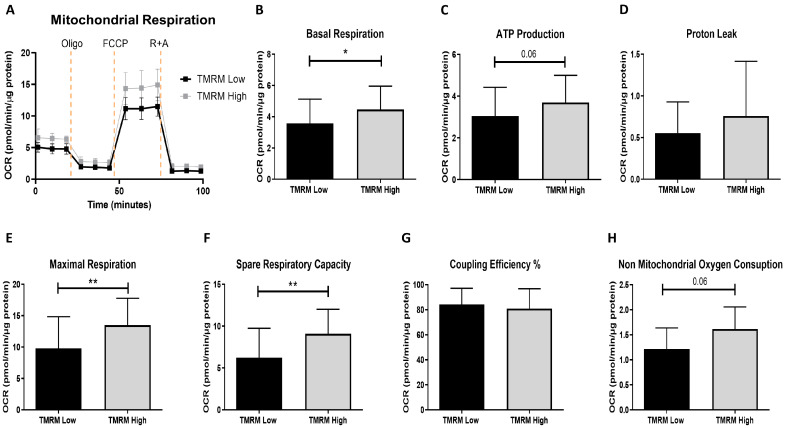
Seahorse extracellular flux analysis for mitochondrial metabolic parameters in TMRM-low and high cells. (**A**) Mitochondrial OCR curves; (**B**) basal respiration; (**C**) ATP production; (**D**) proton leak; (**E**) maximal respiration; (**F**) spare respiratory capacity; (**G**) coupling efficiency (%) and (**H**) non-mitochondrial oxygen consumption. OCR: oxygen consumption rates; Oligo: oligomycin; FCCP: carbonyl cyanide p-trifluoromethoxyphenylhydrazone; R: rotenone; A: antimycin A. Data are represented as mean ± SD. *n* = 5 per group. Statistical differences were calculated significant as * *p* < 0.05, ** *p* < 0.01, determined by Student’s *t*-test (B–G) and by Wilcoxon matched-pairs signed rank test (H).

The bioenergetic profile (Figure 2A) showed that TMRM-high cells had significant higher levels of basal and maximal respiration and spare respiratory capacity (Figure 2B,E,F, respectively). Even if the differences were not significant in both coupled ATP synthesis, proton leak and non-mitochondrial oxygen consumption, there was an increasing trend in TMRM-high cells compared to TMRM-low cells (Figure 2C,D,H). No difference in coupling efficiency could be noticed (Figure 2G). Regarding the energy phenotype, TMRM-high cells were more aerobic than Low, which were more glycolytic (data not shown).

**Figure 3:**
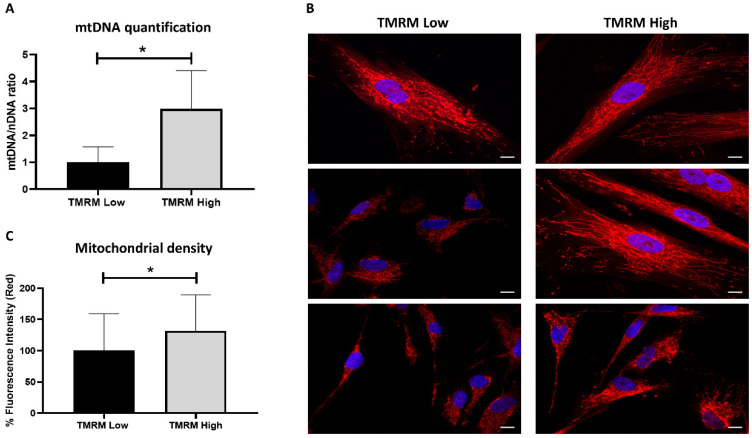
Mitochondrial analysis in TMRM-low and high cells. (**A**) mtDNA content was calculated using quantitative real-time PCR by measuring the ratio of mitochondrially encoded NADH: ubiquinone oxidoreductase core subunit 5 (*ND5*, mitochondrial encoded gene) to β2-microglobulin (*B2M*, nuclear encoded gene) DNA levels. Data are represented as mean ± SD. *n* = 5 per group. Statistical differences were calculated significant as * *p* < 0.05, determined by Student’s *t*-test. (**B**) Representative confocal pictures of MitoTracker Red CMXRos/DAPI to visualize the mitochondrial network. Magnification 63x. Calibration bar 10 µm. (**C**) Percent red fluorescence intensity of MitoTracker staining was calculated using ImageJ software. Data are represented as mean ± SD. Statistical differences were calculated significant as * *p* < 0.05, determined by Student’s *t*-test.

Moreover, mitochondrial DNA was significantly increased in TMRM-high cells compared to low (*n* = 5, mtDNA/nDNA fold increase 1.00 ± 0.58 TMRM-low vs. 2.99 ± 1.42 TMRM-high; *p* = 0.01, Figure 3A). Difference in mtDNA/nDNA ratio is due to changes in mtDNA copy number per cell in relation to mitochondrial density observed in Figure 3C. That reflects difference in mitochondrial biogenesis and not only in mtDNA copy number per mitochondria. To evaluate the mitochondrial dynamics, MitoTracker Red CMXRos was used as a red fluorescent dye that accumulates in living cells with functional mitochondria while nuclei were stained with DAPI. The mitochondrial network was well defined at the perinuclear level, but fluorescence was more diffusely stained throughout the cytoplasm for the high counterparts (Figure 3B).

**Figure 4:**
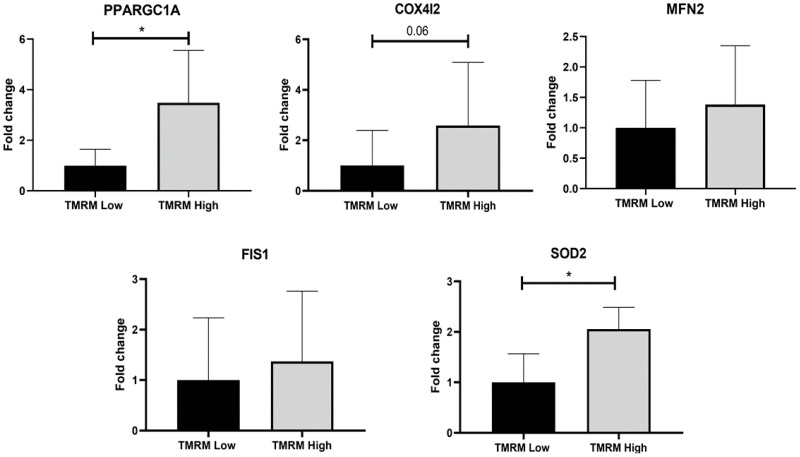
Mitochondrial gene expression in TMRM-low and high cells. mRNA levels were determined by qRT-PCR. *PPARGC1A*: Peroxisome proliferator-activated receptor gamma coactivator 1-alpha; *COX4I2*: cytochrome c oxidase subunit 4I2; *MFN2*: mitofusin 2; *FIS1*: Fission, mitochondrial 1; *SOD2*: superoxide dismutase 2. Data are represented as mean ± SD of the fold change. *n* = 5 per group. Statistical differences were calculated significant as * *p* < 0.05, determined by Student’s *t*-test.

Regarding the gene expression analysis, mitochondrial biogenesis was evaluated by induced expression of peroxisome proliferator-activated receptor gamma coactivator 1-alpha (*PPARGC1A*) (PGC-1α) and *COX4I2*, as an isoform of the cytochrome c oxidase subunit 4. *COX4I2* is one of the nuclear-coded polypeptide chains of cytochrome c oxidase, which expression is controlled by *PPARGC1A*. Thus, increased expression of both genes was observed in TMRM-high samples compared to low cells (Figure 4), especially of *PPARGC1A* (*n* = 5; *PPARGC1A/B2M* fold increase 1.00 ± 0.64 TMRM-low vs. 3.48 ± 2.07 TMRM-high; *p* = 0.04). The antioxidant enzyme *SOD2* expression was higher in TMRM-high cells than in low (Figure 4), in relation with the increased biogenesis observed (*n* = 5; *SOD2/B2M* fold increase 1.00 ± 0.57 TMRM-low vs. 2.05 ± 0.43 TMRM-high; *p* = 0.02). Even if the differences were not significant in both *MFN2* and in *FIS1*, there was a very weak increasing trend in TMRM-high cells compared to TMRM-low cells, in accordance with an impact on mitochondrial dynamics and oxidative metabolism for the cells with high mitochondrial membrane potential (Figure 4).

### 2.3. Gene Expression in Basal Conditions

**Figure 5:**
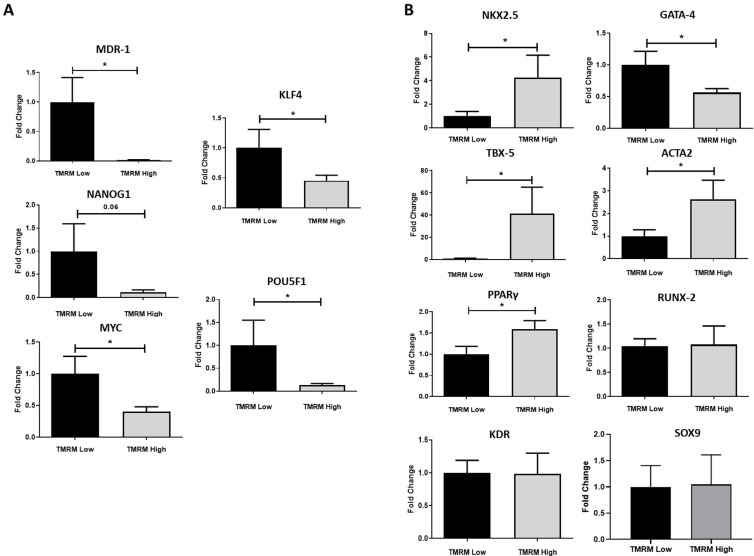
Gene expression of TMRM-low and high cells in basal condition. mRNA expression of markers associated to undifferentiated cells (**A**) and lineage specific cells (**B**) were determined by qRT-PCR. *MDR-1*: multidrug resistance 1; *POU5F1*: POU domain, class 5, transcription factor 1; *NANOG1*: homeobox protein NANOG; *KLF4*: Krüppel-like factor 4; *NKX2.5*: NK2 homeobox 5; *TBX5*: T-Box transcription factor 5; *PPARγ*: peroxisome proliferator-activated receptor gamma; *RUNX-2*: runt-related transcription factor 2; *KDR*: kinase insert domain receptor; *ACTA2*: actin alpha 2, smooth muscle; *SOX9*: SRY-box transcription factor 9. Data are represented as mean ± SD of the fold change. *n* = 4/5 per group. Statistical differences were calculated significant as * *p* < 0.05, determined by paired Student’s *t*-test.

After the isolation of TMRM-low and high cells by sorting, we evaluated if the two cell populations possess transcriptomic differences in growth conditions, analyzing stem cell markers expression (Figure 5A) and their differentiation potential predisposition (Figure 5B). In particular, to assess the degree of stemness of the two populations, we investigated the presence of pluripotency markers, such as octamer-binding transcription factor (Oct-4) (*POU5F1*), Krüppel-like factor 4 (*KLF4*), Nanog (*NANOG1*), and c-Myc (*MYC*), and the marker related to the ABC transporters multidrug-resistance 1 (*MDR-1*), inherently expressed in a wide variety of stem cells. *B2M* gene expression was used as reference. We found a higher expression of all the analyzed stem markers in TMRM-low vs. TMRM-high cells (*n* = 5; *MDR-1/B2M* fold change 1.00 ± 0.41 TMRM-low vs. 0.01 ± 0.007 TMRM-high; *p* = 0.02; *POU5F1/B2M* fold change 1.00 ± 0.55 TMRM-low vs. 0.13 ± 0.03 TMRM-high; *p* = 0.04; *KLF4/B2M* fold change 1.00 ± 0.31 TMRM-low vs. 0.45 ± 0.09 TMRM-high; *p* = 0.04; *MYC/B2M* fold change 1.00 ± 0.27 TMRM-low vs. 0.40 ± 0.07 TMRM-high; *p* = 0.02; Figure 5A).

Conversely, TMRM-high cells were more predisposed to express markers associated to a defined differentiation program (Figure 5B). They expressed higher levels of early cardiac markers *NKX2.5* and *TBX5* (*NKX2.5/B2M* fold change 1.00 ± 0.39 TMRM-low vs. 4.27 ± 1.88 TMRM-high; *p* = 0.05; *TBX5/B2M* fold change 1.00 ± 0.33 TMRM-low vs. 41.29 ± 23.85 TMRM-high; *p* = 0.05). Interestingly, according to tissue hCmPC origin, the expression of *GATA-4*, a very early gene of cardiac commitment, was more expressed in TMRM-low vs. high cells population (*n* = 5; *GATA4/B2M* fold change 1.00 ± 0.21 TMRM-low vs. 0.56 ± 0.06 TMRM-high; *p* = 0.03); [27,28]. The main regulator of adipogenic differentiation *PPARγ* was significantly upregulated in TMRM-high cells (*n* = 5; *PPARγ/B2M* fold change 1.00 ± 0.19 TMRM-low vs. 1.59 ± 0.20 TMRM-high; *p* = 0.03), as well as the smooth muscle marker αSMA *(ACTA2)* (*n* = 5; *ACTA2/B2M* fold change 1.00 ± 0.28 TMRM-low vs. 2.62 ± 0.84 TMRM-high; *p* = 0.03). No differences were detected for osteogenic (*RUNX-2*), chondrogenic (*SOX9*) and endothelial (*KDR*) markers in TMRM-high vs. Low populations (*n* = 4/5).

### 2.4. Multilineage Cardiovascular and Mesenchymal Commitment

To determine whether the different gene expression profile of TMRM-low and high cells correlate to a distinct differentiation capability, cardiovascular and mesenchymal commitment of TMRM-high and low cells was investigated by functional assays, flow cytometry, qRT-PCR, and Western blot.

#### 2.4.1. Endothelial Differentiation

**Figure 6:**
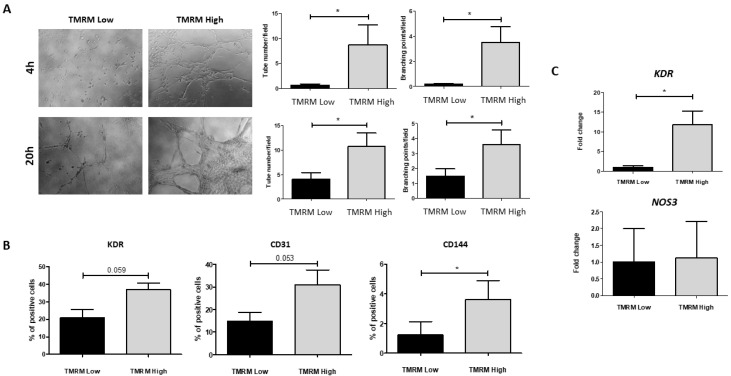
In vitro endothelial differentiation in TMRM-low and high cells. (**A**) Cultrex assay: representative images of tubular-like structures formation in TMRM-low and TMRM-high cells on Cultrex membrane. The left panels show the quantification of tubular-like structures per microscopic field at 4 h and 20 h. The right panels show the number of branching points between tubular-like structures at 4 h and 20 h. In these conditions (*n* = 6); (**B**) FACS analysis: bar graphs show the expression of VEGFR-2/KDR, CD31, and vascular endothelial (VE)-cadherin/CD144 in TMRM-low and TMRM-high cells after culture into endothelial growing medium-2 (EGM-2) for 1 week. In these conditions (*n* = 3); (**C**) gene expression: bar graphs show the expression of VEGFR-2/*KDR* and *NOS3* in TMRM-low, and TMRM-high cells after culture in EGM-2 for 1 week. Data are represented as mean ± SD of the fold change. Statistical differences were calculated significant as * *p* < 0.05, determined by Student’s *t*-test.

Endothelial phenotypes were first investigated by plating TMRM-high and low cells onto Cultrex basement membrane to allow formation of tubular-like structures (Figure 6A). Subsequently, the culture of TMRM-high and low cells in endothelial growing medium-2 (EGM-2) was used to assess their ability to differentiate into mature endothelial cells. Results of these experiments showed that TMRM-high cells were committed to endothelial cell phenotype and that they formed significant more tubular structures compared to TMRM-low cells both at 4 h (*n* = 5; tubular-like structures/field 0.58 ± 0.28 TMRM-low vs. 8.73 ± 3.99 TMRM-high; *p* = 0.049; *n* = 6; branching points/field 0.17 ± 0.06 TMRM-low vs. 3.49 ± 1.28 TMRM-high; *p* = 0.02) and at 20 h (*n* = 6; tubular-like structures/field 4.09 ± 1.28 TMRM-low vs. 10.75 ± 2.74 TMRM-high; *p* = 0.02; branching points/field 1.49 ± 0.48 TMRM-low vs. 3.57 ± 1.00 TMRM-high; *p* = 0.04); in addition, the culture in EGM-2 for 1 week caused a significant increase of the endothelial marker CD144 (*n* = 3; % positive cells 3.62 ± 2.19 TMRM-high vs. 1.23 ± 1.53 TMRM-low; *p* = 0.01) and enhanced the expression, although not significantly, of the endothelial markers kinase insert domain receptor (KDR) and CD31 in TMRM-high vs. low cells, as assessed by flow cytometry (Figure 6B). In addition, the pro-endothelial media induced a significant higher expression of mRNAs for *KDR* in TMRM-high vs. low cells (*n* = 3; 11.79 ± 7.05 TMRM-high vs. 1.00 ± 0.66 TMRM-low), while endothelial nitric oxide synthase (eNOS) (*NOS3*) was unchanged (Figure 6C).

#### 2.4.2. Cardiomyogenic Differentiation

**Figure 7:**
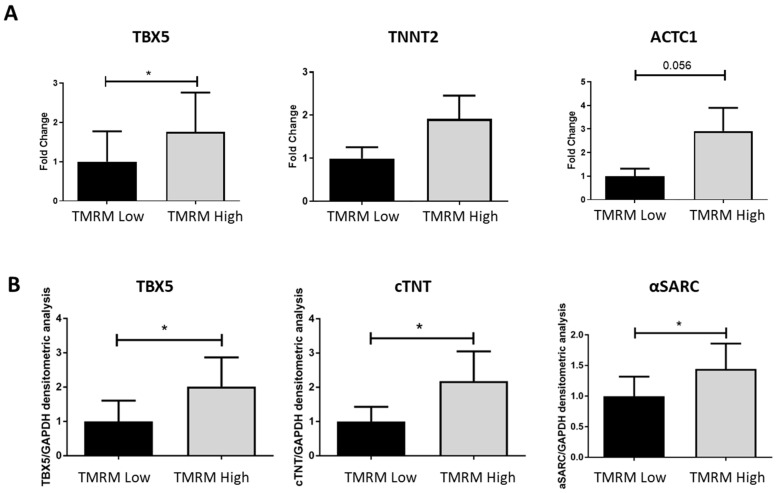
In vitro cardiac differentiation of TMRM-low and high cells. Expression of early cardiac gene/protein: T-Box transcription factor 5 (*TBX5*) and late cardiac genes cTNT (*TNNT2*) and α-sarcomeric actin (*ACTC1*) in TMRM-low and high cells after 1 week of culture in cardiomyogenic differentiation media. The expression of these genes was analyzed by qRT-PCR (*n* = 5) (**A**), and Western blot (*n* = 4) (**B**) Data are represented as mean ± SD of the fold change. Statistical differences were calculated significant as * *p* < 0.05, determined by paired Student’s *t*-test.

To analyze the predisposition of TMRM-low and high to differentiate toward a cardiomyogenic phenotype, we cultured the two populations in cardiac differentiation medium for 7 days and we evaluated the expression of early and late cardiomyogenic markers. We found a general trend of higher expression of T-box transcription factor 5 (*TBX5*), cardiac troponin T (cTNT) *(TNNT2)*, and α-Sarcomeric Actin *(ACTC1)*, both at genes (Figure 7A) and protein (Figure 7B) levels, in the high population. Particularly, the gene expression of the early cardiomyogenic marker *TBX5* resulted significantly upregulated in TMRM-high cells after 7 days of culture (*n* = 5; *TBX5/B2M* fold change 1.00 ± 0.77 TMRM-low vs. 1.76 ± 0.99 TMRM-high; *p* = 0.04). Only a trend of increase expression was reported for the late cardiomyogenic genes *TNNT2* and *ACTC1* at this time-point. In the same culture conditions, the expression of the proteins TBX5, cTNT, and alpha sarcomeric actin (α-SARC) resulted significantly higher in the TMRM-high populations, if compared to the TMRM-low counterpart (*n* = 4; TBX5/GAPDH densitometric analysis 1.00 ± 0.60 TMRM-low vs. 2.01 ± 0.85 TMRM-high; *p* = 0.04; cTNT/GAPDH densitometric analysis 1.00 ± 0.43 TMRM-low vs. 2.18 ± 0.86 TMRM-high; *p* = 0.05; α-SARC/GAPDH densitometric analysis 1.00 ± 0.32 TMRM-low vs. 1.44 ± 0.41 TMRM-high; *p* = 0.02).

#### 2.4.3. Adipogenic Differentiation

**Figure 8:**
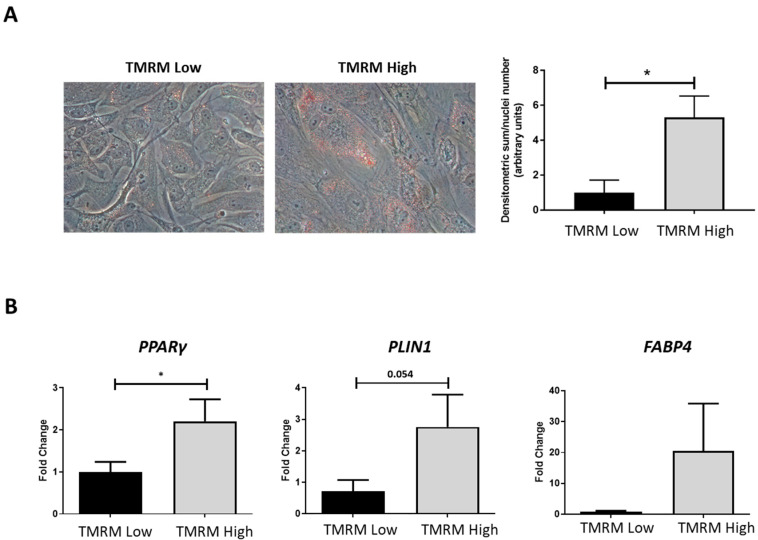
In vitro adipogenic differentiation of TMRM-low and high cells. (**A**) The culture in pro-adipogenic conditions for 1 week favored morphological differentiation in particular of TMRM-high cells, as evidenced by accumulation of lipid droplets in the cytoplasm, stained by Oil-Red-O (left panels). The bar graph shows the relative quantification (right panel). (**B**) Expression of the pro-adipogenic genes *PPARγ*: peroxisome proliferator-activated receptor gamma, *PLIN1*: perilipin 1, and *FABP4*: fatty acid-binding protein 4. Data are represented as mean ± SD of the fold change. *n* = 4 per group. Statistical differences were calculated significant as * *p* < 0.05, determined by Student’s *t*-test.

The ability to differentiate into osteoblasts, adipocytes and chondroblasts is one of the major criteria to recognize cells with MSC phenotype [29]. Due to the mesenchymal nature of hCmPCs, we decided to test the capability of TMRM-low and high cells to differentiate into adipocytes. Thus, we cultured TMRM-low and high in adipogenic conditions for 1 week. The Oil Red O (ORO) staining, to detect the intracellular neutral lipid accumulation, revealed higher lipid accumulation in the TMRM-high population (*n* = 4; densitometric sum/nuclei number 1.00 ± 0.72 TMRM-low vs. 5.31 ± 1.22 TMRM-high; *p* = 0.02; Figure 8A). In agreement with lipid accumulation levels, the expression of the adipogenic genes *PPARγ* was significantly upregulated in the TMRM-high population (*n* = 5; *PPARγ*/*B2M* fold change 1.00 ± 0.24 TMRM-low vs. 2.21 ± 0.52 TMRM-high; *p* = 0.03; Figure 8B). Moreover, we detected a trend of higher expression of *PLIN1* and *FABP4* in TMRM-high cells.

#### 2.4.4. Chondrogenic Differentiation

**Figure 9:**
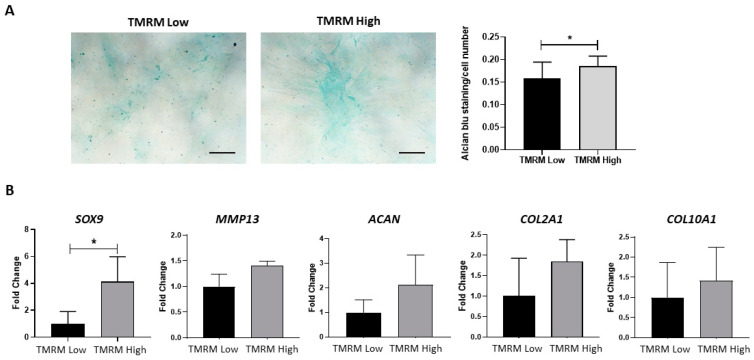
In vitro chondrogenic differentiation of TMRM-low and high cells. (**A**) The culture in pro-chondrogenic conditions for 2 weeks favored increased in sGAG synthesis, as evidenced by Alcian blue staining (left panel). Bar graph shows the relative quantification (right panel). (**B**) Expression of early and late genes involved in chondrogenesis *SOX9*: SRY-box transcription factor 9, *MMP13*: matrix metallopeptidase 13, *ACAN*: aggrecan, *COL2A1*: collagen type II alpha 1 chain and *COL10A1*: collagen type X alpha 1 chain. *n* = 3 per group. Data are represented as mean ± SD of the fold change. *n* = 3 per group. Statistical differences were calculated significant as * *p* < 0.05, determined by Student’s *t*-test.

The chondrogenic differentiation capacity of hCmPC was analyzed by culturing cells in monolayer (2D culture) and under 3D conditions in chemically-defined serum-free media containing TGF-β. The cells were differentiated for two weeks in adherent 2D culture condition to evaluate sGAG by Alcian blue staining, and the 3D high-density pellet culture to analyze gene expression levels. The quantification of Alcian blue staining demonstrated a significant increase in sGAG synthesis in TMRM-high vs. TMRM-low populations (Figure 9A). Moreover, after 14 days, we found a significant upregulation of the early chondro/osteo transcription factor sex-determining region y (SRY)-box transcription factor 9 (*SOX9*) (*n* = 3; *SOX9*/*B2M* fold change 1.00 ± 1.57 TMRM-low vs. 4.14 ± 3.20 TMRM-high; *p* = 0.04; Figure 9B) and a higher expression, although not significant, of *COL2A1*, *COL10A1* as well as *ACAN* and *MMP13* genes involved in chondrogenesis (Figure 9B) in TMRM-high vs. TMRM-low populations.

#### 2.4.5. Osteogenic Differentiation

**Figure 10:**
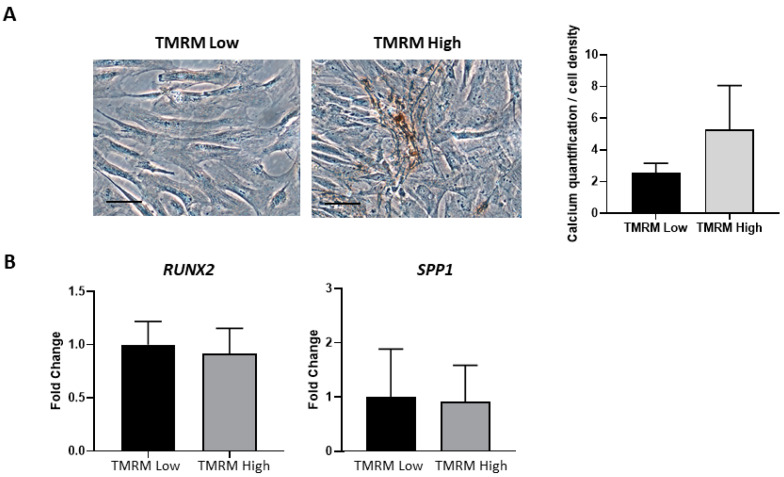
In vitro osteogenic differentiation of TMRM-low and high cells. (**A**) The culture in pro-osteogenic conditions for 1 week enhance the expression of calcium salts, although not significantly (left panel). The bar graph shows the relative quantification (right panel). (**B**) Expression of genes involved in osteogenesis *RUNX2*: runt-related transcription factor 2, *SPP1*: secreted phosphoprotein 1. Data are represented as mean ± SD of the fold change. *n* = 3 per group. Statistical differences were determined by Student’s *t*-test.

TMRM-low and high cells were cultured in osteogenic differentiation media. Results showed similar morphological differentiation of TMRM-low into osteoblast compared with TMRM-high population, although the latter showed, in von Kossa staining, a tendency to accumulate more calcium salts as shown by the increase in intensity of the dark brown metallic silver staining (Figure 10A). In addition, the analysis of genes implicated in osteogenic differentiation, *RUNX2* and *SPP1,* revealed no transcriptional differences between TMRM-low and high populations, as shown immediately after sorting (Figure 5B).

### 2.5. Single Cell Cloning

**Figure 11:**
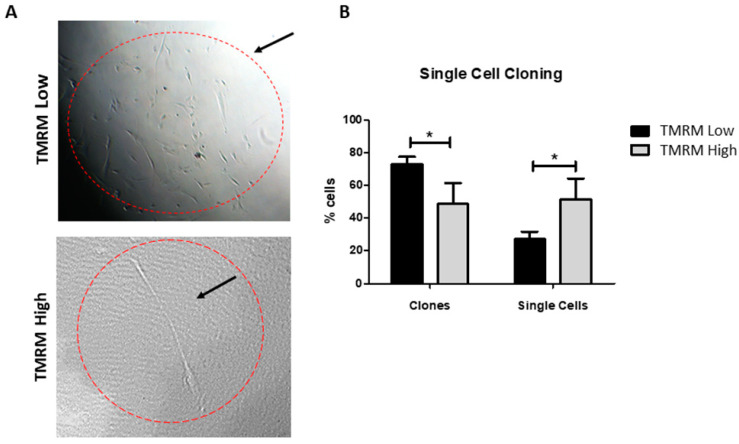
Single cell cloning of TMRM-low and high cells. Representative light microscopy images of TMRM-low and high cells after 1 week of single cells cloning assay (left panel). Bar graph identify the numbers of clones and single cells present after 1 week of culture for both the considered populations. Data are represented as mean ± SD. *n* = 3 per group. Statistical differences were calculated significant as * *p* < 0.05, determined by Student’s *t*-test.

To establish whether the lower differentiation capability of TMRM-low cells could be due to a different commitment state (i.e., early committed cells vs. transit amplifying cells), instead of a senescent state, we performed a single cell cloning assay. The results demonstrated that TMRM-low population are more prone to produce clones vs. TMRM-high cells (Figure 11). Moreover, we analyzed the expression of genes involved in cell cycle regulation *CDKN2A* (p16), *CDKN1A* (p21), and *CDKN1B* (p27), demonstrating that TMRM-low cells expressed significantly lower levels than cells undergoing replicative senescence, used as a control (*n* = 3; *p16/B2M* fold change 0.07 ± 0.02 TMRM-low vs. 1 ± 0.41 senescent cells; *p21/B2M* fold increase 0.42 ± 0.27 TMRM-low vs. 1 ± 0.32 senescent cells; *p27/B2M* fold change 0.62 ± 1.14 TMRM-low vs. 1 ± 0.26 senescent cells; Supplementary Figure S2).

### 2.6. TMRM Transition in Culture and During Differentiation

**Figure 12:**
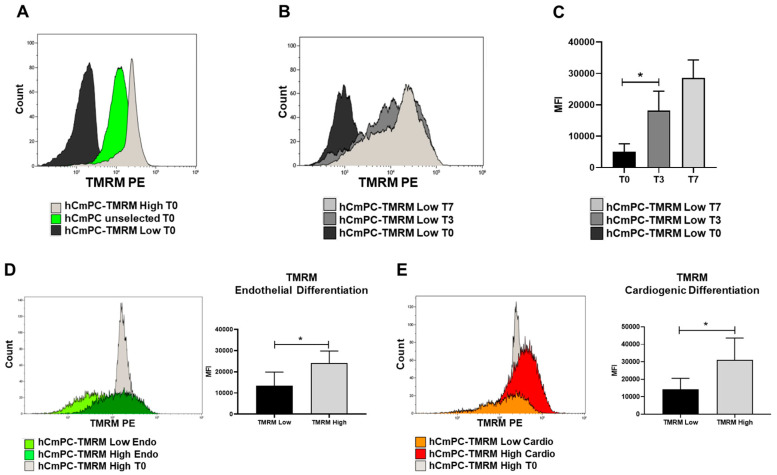
TMRM transition in culture and during differentiation. (**A**) Fluorescence levels of TMRM in the hCmPC population: before sorting (green histogram) and after selection to separate the population with the highest levels of TMRM (high, light grey histogram) from the population expressing the lowest levels of TMRM (low, black histogram). (**B**) Histogram plot of the transition level of TMRM expression in TMRM-low population in a time course experiment from T0 to T7 and its relative quantification (**C**). (**D**,**E**) Histogram plot and quantification of TMRM expression levels shown as MFI in TMRM-low and high population after endothelial (**D**) and cardiomyogenic (**E**) differentiation. Data are represented as mean ± SD. *n* = 3 per group. Statistical differences were calculated significant as * *p* < 0.05, determined by Student’s *t*-test.

After TMRM sorting (Figure 12A), we investigated the mean fluorescence intensity (MFI) of TMRM to assess if TMRM-low cells pass through a TMRM-high state in culture before turning on the differentiation program. After 0, 3, and 7 days in TMRM-low cells, the results showed a progressive increment of MFI levels that reached the significance at T3 when compared to the starting point (Figure 12B,C). Then, we focused our attention on endothelial (Figure 12D) and cardiomyogenic (Figure 12E) differentiation to evaluate differences in terms of MFI of TMRM in both subpopulations. We confirmed higher MFI levels in TMRM-high than in low cells after 1 week of differentiation (Figure 12D,E). To note, TMRM-high population displayed higher level of MFI when compared to their undifferentiated counterpart (histogram grey in Figure 12D,E) in line with a greater number of mitochondria (data not shown).

## 3. Discussion

The importance of mitochondrial metabolism in mediating stem cell activity is becoming increasingly recognized [30]. These cells are subjected to the decisions of self-renew or differentiation coupled to highly regulated molecular and metabolic events. Thus, defining the involvement of mitochondria in cell fate is needed.

Here, by TMRM sorting, we isolated two distinct cell populations characterized by similar antigen mesenchymal expression marker. In line with a more immature phenotype, TMRM-low cells displayed a slightly higher expression of stem cells cardiac marker CD117 (c-kit). C-kit CSCs are known to constitute the more primitive mesenchymal sub-population [10] exhibiting a greater capacity for endodermal differentiation and enhanced multilineage differentiation efficiency [31,32]. Thus, the expression of c-kit (a type III receptor tyrosine kinase) seems necessary. Indeed, c-kit haploinsufficiency in c-kit-deficient mice induced a falling myocardial repair after injury [33]. Furthermore, change of c-kit gene for Cre Recombinase expression in Cre/Lox genetic fate map animal models induced a detrimental c-kit haploinsufficiency that avoids efficient labeling of true CSCs and affects the regenerative capacity of these cells [34]. To sum up, the adult c-kit-labeled CSCs are strongly myogenic and the adult myocardium depends on c-kit expression to regenerate after injury and to counteract the cardiac effects of aging [35].

In our study, TMRM-low hCmPCs presented a significant lower spare respiratory capacity associated to a reduction in the mitochondrial contribution to ATP synthesis as indicators of ‘‘stemness’’ or glycolysis-based anabolic requirements for less differentiated cells that could evolve towards differentiation or senescence as previously reported [23,36,37]. Our results on single cell cloning and comparison with cells undergoing replicative senescent, excluded senescence in low hCmPCs cells. Data on maximal respiration reinforced the conclusion that the contribution of mitochondria to oxygen consumption in TMRM-low and high cells was both diverse and distinct. Cells with low Δψm had lower maximal respiratory and spare capacities than cells with high Δψm, which could indicate decreased oxidative substrate availability or changes in mitochondrial mass or integrity. As previously described for other MSCs, the capacity for High Δψm hCmPCs to respond to oxidative stress or increased energy demand was relevant [38,39]. Consistent with our data, this was related to high induction of SOD2 expression in High Δψm cells.

This bioenergetic reserve capacity is also influenced by overall mitochondrial mass, activity of mitochondrial complexes and mitochondrial network dynamics related to mitochondrial fusion/fission [40]. Due to the processes of fusion and fission, also the quantity of mitochondria can change in a dynamic manner. Lack of fusion-associated proteins, such as optic atrophy 1 (Opa1), mitofusin 1 and mitofusin 2 (MFN1 and MFN2) is sufficient to cause dysfunction of mitochondrial fusion due to the fact that mitochondria are fragmented, and their numbers are increased. However, the loss of either dynamic-related protein 1 (Drp1) or fission 1 (FIS1), both fission-related proteins, causes mitochondria to elongate and form long, continuous tubules [41]. In addition, mitochondria with lower membrane potential have no or little fusion capacity [42,43]. In our study, the TMRM-low and high cells showed difference in mitochondrial network distribution in the cytoplasm supporting changes in mitochondrial dynamic equilibrium.

Knowing that metabolic switch during differentiation is dependent on increased mitochondrial activity [22], we have looked for mitochondrial biogenesis. We demonstrated an increase in cells with high Δψm of cytochrome c oxidase subunit 4I2 (COX4I2) and PGC-1α. In hCmPCs, the upregulation of the mitochondrial biogenesis regulator PGC-1α and anti-oxidant enzymes such as SOD2 were associated to a marked increase in mitochondrial mass and oxygen consumption as previously shown for osteogenic differentiation [44].

Therefore, the hCmPCs with high Δψm showed several features common to cells that are going to differentiate as we showed by cell fate biomarkers. An induction of efficient oxidative metabolism was proved to be an early event in hCmPCs prior to membrane and functional events of cell fate. In agreement with data of other groups, who showed that when the mtDNA copy number was analyzed during differentiation, a dynamic change, we demonstrated that High TMRM population have greater mitochondria content than their more stem counterpart. Moreover, both population were characterized by a progressive shift toward a higher Δψm during differentiation. One explanation for this could be that down-regulation of glycolysis genes and activation of respiratory genes by PGC-1α increased the mtDNA [45,46]. In addition, increased oxygen consumption and oxidative ATP synthesis are dependent on increased mtDNA copy number [47].

Taking into account that the transition from glycolysis to mitochondrial oxidative metabolism and maintenance of mitochondrial electron transport function are critical for differentiation [48,49], our results suggest that cells with low Δψm rely mostly on glycolysis to meet their energy demands, instead the cells with high Δψm prefer the mitochondrial respiration. In light of these findings, mitochondrial metabolism is no longer considered a consequence of cellular differentiation, but rather a key regulatory mechanism of this process as previously reported [50]. Indeed, the increase of TMRM fluorescence during differentiation could represent a sign of mitochondria increment in the cell [51]. Based on our evidence we can propose that a metabolic shift is required also for TMRM-low cells to begin the differentiation. Thus, in a physiological context, all hCmPC are able to differentiate, but some (TMRM-high cells) are quickly “mobilized” with a better efficiency during the damage, whereas others (TMRM-low cells, which never reached high MFI level) represent the reservoir. We could conclude that oxidative metabolism is necessary for differentiation and cells with high Δψm present higher capacity of differentiation.

Numerous data suggest that the main reparative mechanism of both hCmPCs and other MSCs rely on the release of paracrine factors (cytokines, growth factors, microRNAs, and exosomes) [52] rather than direct differentiation [11,12] of the injected cells. However, it should be taken into account that the mechanism of action could reflect the methods of cell delivery, as they could influence the success of the therapy if improperly applied [16].

The low engraftment rate is another issue for the limited effect of stem cell therapies on cardiac repair. In this view, our results could have important and immediate therapeutic applications in enhancing engraftment of hCmPCs. Indeed, based on mitochondrial membrane potential (Δψm), we isolated two cell populations with distinct metabolic and stemness features that could find application in different clinical settings. Speculatively, the TMRM-low cells, characterized by higher clonogenic and self-renewing activity, could longer persist in the damaged area, as seen for other cell types [24]. Indeed, this subpopulation which mostly relies on the anaerobic process of glycolysis for energy production, could survive longer in the hypoxic conditions typical of damaged cardiac areas as for instance the border zone of myocardial infarction [53]. On the other hand, TMRM-high cells, as hCmPCs subpopulation with a more advanced commitment level, may participate to cardiac cell replacement and homeostasis retrieval [54].

In conclusion, our work provides the first detailed characterization of energy and mitochondrial metabolism of hCmPCs with differential Δψm, defining two subpopulations of either stem-like or pre-committed cells. These findings pave the way to future regenerative cardiac medicine options and disease modelling [55].

## 4. Materials and Methods

### 4.1. Ethics Statement

hCmPCs were isolated from 6 cadaveric donors obtained from Fondazione Banca dei Tessuti di Treviso (MTA n° 257/A1/2016). Cadaveric donors were selected from subject who died from causes not related to heart disease and who did not present in life risk factors or concomitant diseases that could affect the quality of isolated hCmPCs.

### 4.2. Cell Culture and TMRM Sorting

Specimens from right atrial appendages were obtained from all the donors. The isolated myocardial tissue was cut into 1 to 2 mm^3^ pieces, washed with phosphate-buffered saline (PBS; Lonza, Milan, Italy) and digested 4 times at 37 °C in a 3 mg/mL collagenase solution (Serva, Heidelberg, Germany). After digestion, the solution was filtered using 70 μm mesh nylon filters (BD Biosciences, Milan, Italy). Cell suspension were finally seeded into uncoated Petri dishes (Corning, Turin, Italy) containing Ham’s F12 medium (Lonza, Milan, Italy) supplemented with 10% fetal bovine serum (FBS; Thermo Fisher Scientific, Milan, Italy), 2mM L-Glutathione (Sigma-Aldrich, Milan, Italy), 5 × 10^3^ U/mL human erythropoietin (Sigma-Aldrich, Milan, Italy), 10 ng/mL basic fibroblast growth factor (bFGF; PeproTech, Neuilly-Sur-Seine, France), and antibiotics (Lonza, Milan, Italy). Cells were incubated at 37 °C and 5% CO_2_. After 48 h, the medium was completely changed to remove dead non-adherent cells. When hCmPCs reached the 70% of confluence, they were split 1:10 and seeded into 10 cm uncoated Petri dishes (Corning, Turin, Italy) (pre-expansion period). At 70% of confluence, the cells were detached using TrypLE Select (Thermo Fisher Scientific, Milan, Italy) and incubated at 37 °C for 30 min with 100 nM tetramethylrhodamine (TMRM) (Thermo Fisher Scientific, Milan, Italy). After cell resuspension in PBS with 10% FBS, cells were sorted using a FACSAria flow cytometer/cell sorter (BD Biosciences, Milan, Italy) to identify and separate TMRM-high from TMRM-low subpopulations. Sorting setup, based on the scatter and fluorescent parameters, and appropriate gating, to exclude dead and double cells, were established using unlabeled cells. For a schematic representation, see Supplementary Figure S1.

### 4.3. FACS Analysis

TMRM-high and TMRM-low hCmPCs cultured in growth conditions were characterized for surface marker expression by FACS analysis. The cells were detached with TrypLE Select (Thermo Fisher Scientific, Milan, Italy) and resuspended in PBS containing 0.1% bovine serum albumin (BSA; Gibco) and 2mM ethylenediaminetetraacetic acid (EDTA; Gibco, Thermo Fisher Scientific, Milan, Italy). Then, they were incubated with fluorochrome-conjugated monoclonal antibodies in the dark for 15 min at room temperature (RT). After washing, cells were centrifuged for 10 min at 400× *g* at 4 °C to remove unbound antibodies and resuspended in 250 µL of washing buffer. Then, they were analyzed by flow cytometry (Gallios, Beckman Coulter, Villepinte, France). The antibodies used are the following: nerve growth factor receptor (NGFR)-PE (clone C40-1457; BD PharMingen, BD Biosciences, Milan, Italy), Lin-FITC (cocktail of biotin-conjugated monoclonal antibodies against CD2, CD3, CD11b, CD14, CD15, CD16, CD19, CD56, CD123, and CD235a + anti-biotin FITC clone Bio3-18E7, Miltenyi Biotec, Bologna, Italy), KDR-PE (clone 89106; R&D Systems, Milan, Italy), CD105-APC (clone 266; BD PharMingen, BD Biosciences, Milan, Italy), CD90-FITC (clone 5E10; BD PharMingen, BD Biosciences, Milan, Italy) and CD117-APC (clone YB5.B8; BD PharMingen, BD Biosciences, Milan, Italy). The proper isotype control was used as reference. To study TMRM transition, TMRM-high and TMRM-low hCmPCs were incubated at 37 °C for 30 min with 100 nM TMRM, then detached and analyzed by BD FACS Gallios (BD Bioscience, Milan, Italy). Five thousand events were acquired on a BD FACS Gallios. Data were analyzed with Kaluza Software (Beckman Coulter, Brea, CA, USA) and reported as percentage of positive cells (Figure 1 and Figure 6) or as MFI (Figure 12).

### 4.4. Seahorse Extracellular Flux (XF) Cell Mito Stress Test

To assess mitochondrial cell respiration, a Seahorse XFe24 Apparatus (Seahorse, Agilent Technologies, Santa Clara, CA, USA) was used. hCmPC TMRM-low and TMRM-high cells were seeded on XF Cell Culture Microplates (Seahorse, Agilent Technologies, Santa Clara, CA, USA) at a concentration of 1.0 × 10^5^/well in 200 μL culture medium and incubated overnight at 37 °C. Culture medium was removed one hour before the test and replaced by 500 μL of pre-warmed un-buffered DMEM supplemented with L-glutamine (2 mM), sodium pyruvate (1 mM) and glucose (10 mM), pH 7.4. The culture plate was incubated in a non-CO_2_ incubator at 37 °C (O_2_ 20–21%). The Agilent Seahorse XF cell Mito Stress Test measures the key parameters of mitochondrial respiration, using specific mitochondrial inhibitors and uncouplers (all from Sigma-Aldrich, St. Louis, MO, USA). Oligomycin (1 μM), carbonyl cyanide-p-trifluoromethoxyphenylhydrazone FCCP (1 μM), rotenone, and antimycin A combination (1 μM each) were injected, subsequently, into each well following the manufacturer’s recommendations. Basal level of oxygen consumption (OCR) was first measured followed by oligomycin injection to inhibit ATP synthase and then FCCP, as a respiratory chain uncoupler, was injected to determine the maximal oxygen consumption rate and calculate mitochondrial ATP level. Finally, a mixture of rotenone/antimycin A was distributed to inhibit electron flux through complexes I and III, shutting down mitochondrial respiration to determine the proton leak. All measurements were normalized to the protein content (μg) of each well using Pierce BCA Protein Assay Kit (Thermo Fisher Scientific, Waltham, MA, USA, Cat. No. 23227).

### 4.5. Mitochondrial DNA Quantification

Total DNA was extracted from hCmPCs with the DNeasy Blood & Tissue Kit (QIAGEN, Valencia, CA, USA, Cat. No. 69504). Quantitative PCR (qPCR) was performed with SYBR Premix Ex Taq (Tli RNase H Plus; Takara, Dalian, China) in triplicate and in 10 μL final volume. Standard curves were established using primers described in Supplementary Table S1 and serial dilutions of the NADH dehydrogenase subunit 5 gene (ND5, mitochondrial genome), and β2-microglobulin (B2M, nuclear genome). Data are expressed as mitochondrial DNA (mtDNA) to nuclear DNA (nDNA) ratio for each sample, calculated by the comparative CT method (2−ΔΔCT) [56].

### 4.6. Mitochondrial Network

Functional status of mitochondria was evaluated by MitoTracker Red CMXRos (Thermo Fisher Scientific, Waltham, MA, USA, Cat. No. M7512) dye, depending on mitochondrial membrane potential of living cells. hCmPCs were seeded in Nunc Lab-Tek chamber slides (Thermo Fisher Scientific, Waltham, MA, USA) at a concentration of 5.0 × 10^4^/well in 500 μL culture medium, incubated overnight at 37 °C and then for 30 min with the MitoTracker Red CMXRos at final concentrations of 200 nM in serum-free medium. After cell fixing in 4% formaldehyde, the fluorescent dye 4′,6-Diamidine-2′-phenylindole dihydrochloride (DAPI) (Sigma-Aldrich, St. Louis, MO, USA, Cat. No. 10236276001) was applied for 6 min (dilution 1:20). Fluorescence of mitochondrial dye was imaged using a Zeiss LSM 780 confocal laser scanning microscope (Zeiss, Oberkochen, Germany) and quantitative analyses were performed using ImageJ software (NIH), expressed as percent (%) fluorescent intensity (red) [57].

### 4.7. Gene Expression Analysis

Gene expression was assessed via quantitative real time polymerase chain reaction (qRT-PCR). Total RNA was isolated using ReliaPrep RNA Cell Miniprep System (Madison, WI, USA, Cat. No. Z6012). cDNA was prepared by High-Capacity cDNA Reverse Transcription Kits (Thermo Fisher Scientific, Waltham, MA, USA, Cat. No. 4368814) and qRT-PCR was performed with SYBR Premix Ex Taq (Tli RNase H Plus; Takara, Dalian, China) in triplicate and 10 μL final volume on a StepOne Real-Time PCR System (Thermo Fisher Scientific, Waltham, MA, USA). β2-microglobulin (*B2M*) was used as reference and primers sequence for qRT-PCR, always encompassing two exons for each gene, are described in Supplementary Table S1. Selected genes corresponded to those involved in mitochondrial biogenesis (*PPARGC1A* and *COX4I2*), mitochondrial dynamics (*MFN2* and *FIS1*) and *SOD2,* as well as those involved in cell lineage from hCmPCs. In basal conditions, we evaluated the expression of stem cell markers (*MDR-1*, *Oct-4*, *c-Myc*, *Nanog,* and *KLF4*), cardiac markers (*GATA4, NKX2.5,* and *TBX5*), adipogenic (*PPARγ*), osteogenic (*RUNKX-2*), endothelial (*KDR*) smooth muscle markers (*α-SMA*), and chondrogenic (*SOX9*). After cardiomyogenic differentiation we assessed the expression of the early cardiac markers (*TBX5*) and late cardiac markers (*cTNT* and *α-SARC*). The adipogenic markers (*PPARγ*, *FABP4,* and *PLIN1*) after adipogenic differentiation, the endothelial markers *KDR* and *NOS3* after endothelial differentiation, the chondrogenic markers (*SOX 9, ACAN, COL2A1, MMP13,* and *COL10A1*) and the osteogenic markers *RUNX2* and osteopontin (*SPP1*) were analyzed. Gene expression was calculated with the 2−ΔΔCT method.

### 4.8. Single Cell Cloning

TMRM-high and TMRM-low hCmPCs were plated in growth medium on 96-well plates, one cell for each well by FACSAria, to generate clones. Each well was scored for the presence or absence of single cells. Wells containing no cells or more than 1 cell were discarded. The medium was changed two times per week. After 7 days, images of each well were obtained using Axiovert 40C microscope supplied with Axiocam 503 (Zeiss, Oberkochen, Germany). The count of clones and single cells normalized on the total cell number was performed.

### 4.9. Endothelial Differentiation

Endothelial commitment was analyzed by culturing TMRM-high and TMRM-low hCmPCs at a density of 30,000 cells/cm^2^ for 1 week in endothelial growth medium-2 (EGM-2, Lonza, Milan, Italy), The Cultrex assay, qRT-PCR, and FACS analysis were performed to check the endothelial phenotype.

### 4.10. Cultrex Assays

To evaluate the capability of TMRM-high and TMRM-low populations to form vascular structures in vitro, immediately after cell sorting, cells were resuspended and diluted to 4 × 10^4^ mL in endothelial growth medium-2 (EGM-2, Lonza, Milan, Italy) and plated on Cultrex basement membrane in 96-well plates. Cells plated in EBM were used as negative control. TMRM-high and TMRM-low populations were observed after 4 h and 20 h. Images were acquired by Axiovert 40C microscope supplied with Axiocam 503 (Zeiss, Oberkochen, Germany). The quantification of tubular-like structures and branching points per field was performed, evaluating at least 10 fields per sample.

### 4.11. Cardiac Differentiation

TMRM-high and TMRM-low hCmPCs were plated at a density of 30,000 cells/cm^2^ for 1 week in a medium consisting of IMDM containing 5% FBS (EuroClone, Milan, Italy), 0.1 U/mL penicillin (Lonza, Italy), 0.1 ug/mL streptomycin (Lonza, Milan, Italy), 1% L-glutamine (Lonza, Milan, Italy), 5 µm All-trans retinoic acid (ATRA, Sigma-Aldrich, Milan, Italy) and 5 μm phenyl-butyrate (Sigma-Aldrich, Milan, Italy) to induce their cardiac differentiation. qRT-PCR and Western blot analysis were performed to check cardiac differentiation marker expression.

### 4.12. Adipogenic Differentiation

TMRM-high and TMRM-low populations were plated at a concentration of 30,000 cells/cm^2^ and cultured in adipogenic conditions for 1 week [58]. The adipogenic medium consists of IMDM supplemented with 10% FBS (Sigma-Aldrich, Milan, Italy), 0.5 mmol/L 3-isobutyl-1-methylxanthine (Sigma-AldrichMilan, Italy), 1 µmol/L hydrocortisone (Sigma-Aldrich, Milan, Italy), 0.1 mmol/L indomethacin (Sigma-Aldrich, Milan, Italy), 10,000 U/mL Penicillin (Thermo Fisher Scientific, Milan, Italy), 10,000 µg/mL Streptomycin (Thermo Fisher Scientific, Milan, Italy) and 20 mmol/L L-Glutamine (Sigma-Aldrich, Milan, Italy). Lipid accumulation was tested by Oil-Red O staining (ORO; Sigma-AldrichMilan, Italy). qRT-PCR for *PPARγ*, *FABP4,* and *PLIN1* (for primers see Supplementary Table S1) were used to check adipogenic mediator expression.

### 4.13. Oil Red O Staining

Oil Red O (ORO) Staining was performed to analyze lipid accumulation in TMRM-high and TMRM-low hCmPCs. After fixation with 4% paraformaldehyde (Santa-Cruz Biotechnology, Inc., Santa Cruz, CA, USA) in PBS, cells were washed in PBS and stained with 1% ORO solution (Fulka) in 60% isopropanol (Sigma-Aldrich, Milan, Italy) for 1 h at RT. After PBS washes to remove the unbound dye, the images were acquired by Axiovert 200M supplied with Axiocam 503 (Zeiss, Oberkochen, Germany) in black and white, using phase H, to highlight black lipid depots. The quantification was performed with the software AxioVision Rel. 4.8, evaluating at least 10 fields per sample.

### 4.14. Chondrogenic Differentiation

TMRM-high and TMRM-low populations were plated at concentration of 30,000 cell/cm^2^ for 2D environment and cultured in chondrogenic condition for 2 weeks [59,60]. For chondrogenic differentiation in high-density pellet cultures 3 × 10^5^ hCmPC TMRM-low and high cells were centrifuged (150× *g*, 5 min) in a 1.5 mL polypropylene tube to form a pellet. The chondrogenic medium consists of DMDM supplemented with 100 nM dexamethasone (Sigma-Aldrich), 50 μM ascorbic acid (Sigma-Aldrich, Milan, Italy), 5 ng/mL TGF-β (PeproTech, Neuilly-Sur-Seine, France) and 10,000 µg/mL streptomycin (Thermo Fisher Scientific, Milan, Italy). For the 3D culture condition, the media was supplemented with 10 ng/mL TGF-β (PeproTech, Neuilly-Sur-Seine, France) instead of 5 ng/mL [61]. sGAG synthesis was tested by Alcian blue staining (Sigma-Aldrich, Milan, Italy). qRT-PCR for *SOX9, ACAN, COL2A1, MMP13*, and *COL10A1* genes were used to check chondrogenic differentiation progression.

### 4.15. Alcian Blue Staining

Alcian blue staining was performed to analyze sGAG synthesis in TMRM-high and TMRM-low hCmPCs after differentiation as reported in [59]. Cells were stained with 1% Alcian blue 8GX (Sigma-Aldrich, Milan, Italy) in 0.1 M HCl overnight, then washed extensively with distilled water to remove any unincorporated dye and viewed under a light microscope (Axiovert 40C microscope supplied with Axiocam 503, Zeiss, Oberkochen, Germany). For quantification analysis, incorporated dye was removed by adding 6M guanidine–HCl for 6 h at room temperature and the optical density of the extracted dye was measured at 620 nm on a spectrophotometer.

### 4.16. Osteogenic Differentiation

Osteogenic differentiation was induced by culturing TMRM-high and TMRM-low cell populations for 1 week in osteogenic differentiation medium consisting of IMDM supplemented with 0.1 μM dexamethasone (Sigma-Aldrich, Milan, Italy), 10 mM β-glycerol phosphate (Sigma-Aldrich, Milan, Italy), and 0.2 mM ascorbic acid (AsA; Sigma-Aldrich, Milan, Italy). The deposition of mineralized matrix was assessed by von Kossa staining. qRT-PCR for *RUNX2* and Osteopontin (*SPP1*) genes were used to check osteogenic differentiation progression.

### 4.17. von Kossa Staining

TMRM-high and TMRM-low hCmPCs were incubated using a 1% silver nitrate solution in the dark for 30 min after fixation in 4% paraformaldehyde (Sigma-Aldrich, Milan, Italy). Then cells were thoroughly washed with distilled water and exposed to UV light for 60 min to visualize the crystals (to detect the presence of calcium deposition in osteocyte precursor). After the exposure, the unbound silver was removed by extensive washing. Finally, the cells were counter-stained using toluidine blue.

### 4.18. Crystal Violet Staining

Crystal violet staining/extraction was used to estimate the cell number and normalized of Alcian blue and von Kossa staining. Cells were stained with 0.1% crystal violet (Sigma-Aldrich, Milan, Italy) for 20 min, washed 3 times with distilled water, aspirate, and allowed to air dry. For quantification analysis, incorporated dye extract by adding 10% acetic acid solution for 20 min with shaking at room temperature and the optical density was measured at 590 nm on a spectrophotometer.

### 4.19. Western Blot

TMRM-high and TMRM-low populations were lysed and treated with protease and phosphatase inhibitor cocktails to extract total proteins. After quantification with DC protein assay (Bio-Rad, Milan, Italy), proteins were run on SDS-PAGE gel (NuPAGE precast 4–12%, Thermo Fisher, Milan, Italy) and transferred to Trans-Blot^®^ Turbo™ nitrocellulose membrane (Bio-Rad) with Trans-Blot^®^ Turbo™ transfer system. The membranes were blocked for 1 h at RT in 5% non–fat dry milk (ChemCruz, Huissen, The Netherlands) in PBS containing 0.05% Tween^®^ 20 (Sigma-Aldrich, Milan, Italy) and then incubated with the appropriate primary antibody at 4 °C overnight. The primary antibodies used are anti-TBX5 (ab137833; Abcam, Cambridge, UK), anti-cTNT (MA5-12960; Thermo Fisher Scientific, Milan, Italy), anti-α-SARC (A2172; Sigma-Aldrich, Milan, Italy). After washes in PBS containing 0.05% Tween^®^ 20 (Sigma-Aldrich, Milan, Italy), the membranes were incubated 1 h at RT with the appropriate HRP-conjugated secondary antibody. Blots were developed using enhanced chemiluminescence Western blotting detection system (GE Healthcare, Milan, Italy) and images acquired and quantified with the Uvitec Cambridge system. The normalization was performed on the housekeeping protein GAPDH (sc-25778; Santa Cruz Biotechnology, Inc., Santa Cruz, CA, USA).

### 4.20. Statistics

Statistical analyses among different groups were performed with GraphPad Prism 8. Normality for continuous variables was assessed by means of the Shapiro–Wilk test. Comparisons between groups were made using two-tailed paired *t*-test or Wilcoxon rank sum test as appropriate.

In the figures, error bars represent SD and according to the GraphPad classification of significance points * *p* < 0.05, ** *p* < 0.01.

## Supplementary Materials

The following are available online at https://www.mdpi.com/1422-0067/21/20/7467/s1.

## Abbreviations

ACAN	Aggrecan
ATP	Adenosine triphosphate
B2M	β2 microglobulin
BM	Bone marrow
c-Myc	MYC
COL2A1	Collagen type II alpha 1 chain
COL10A1	Collagen type X alpha 1 chain
COX4I2	Cytochrome c oxidase subunit 4I2
CPCs	Cardiac progenitor cells
CSCs	Cardiac stem cells
cTNT	Cardiac troponin T
ECAR	Extracellular acidification rate
ESCs	Embryonic stem cells
FABP4	Fatty acid-binding protein 4
FACS	Fluorescent activated cell sorting
FCCP	Carbonyl cyanide-p-trifluoromethoxyphenylhydrazone
FIS1	Mitochondrial fission 1 protein
GATA4	GATA Binding protein 4
hCmPC	Human cardiac mesenchymal progenitor cells
iPSCs	Induced pluripotent stem cells
KDR	kinase insert domain receptor
KLF4	Krüppel like factor 4
MDR-1	Multidrug-resistance
MFN2	Mitofusin-2
MFIM	Mitofusin-2Mean fluorescence intensity
MP13	Matrix metallopeptidase 13
mtDNA	Mitochondrial Deoxyribonucleic Acid
ND5	Mitochondrially encoded NADH, ubiquinone oxidoreductase core subunit 5
nDNA	Nuclear Deoxyribonucleic Acid
NGFR	Nerve growth factor receptor
NKX2.5	NK2 Homeobox 5
NOS3	Endothelial nitric oxide synthase
OCR	Oxygen consumption rate
Oct-4	Octamer-binding transcription factor
Opa1	Optic atrophy type 1
OXPHOS	Oxidative phosphorylation
PCR	Polymerase chain reaction
PGC-1α	Peroxisome proliferator-activated receptor gamma coactivator 1-alpha
PLIN-1	Perilipin 1
PPARγ	Peroxisome proliferator-activated receptor gamma
ROS	Reactive oxygen species
RUNX-2	Runt-related transcription factor 2
SPP1	Secreted Phosphoprotein 1
SOD2	Superoxide dismutase 2
SOX9	SRY-Box transcription factor 9
TBX5	T-Box transcription factor 5
TMRM	Tetramethylrhodamine methyl ester
VEGFR-2	Vascular endothelial growth factor receptor 2
α-SARC	Alpha sarcomeric actin
α-SMA	Alpha-smooth muscle actin
Δψm	Mitochondrial membrane potential
