# Supplementary material for: Differences in Mitochondrial Membrane Potential Identify Distinct Populations of Human Cardiac Mesenchymal Progenitor Cells
Source: Int J Mol Sci. 2020 Oct 10;21(20):7467. doi: 10.3390/ijms21207467 (PMC7590175; doi:10.3390/ijms21207467)
Supplement: Supplementary file 1 [file ijms-21-07467-s001.pdf]

Supplementary Figures and Table:

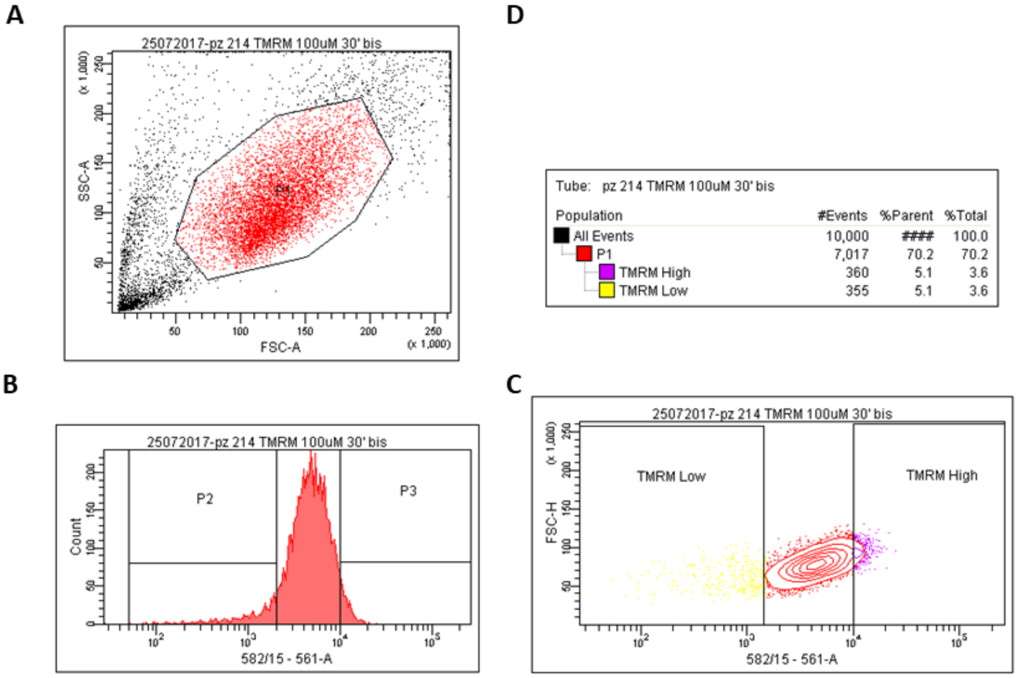

Supplementary Figure S1. Sorting procedure for hCmPCs. A hierarchical gating strategy was adopted; **(A)** shows the initial gating that was established on the scatter plot to identify cells with appropriate dimensions and complexity within the unselected hCmPC cells. **(B-C)** The appropriate gating to separate TMRM Low from TMRM High cells was established each time using phycoerythrin fluorescence intensity to sorted populations with lowest 5% (TMRM Low in gate P2) or highest 5% mitochondrial membrane potential (TMRM High in gate P3) as reported in panel **(D).**

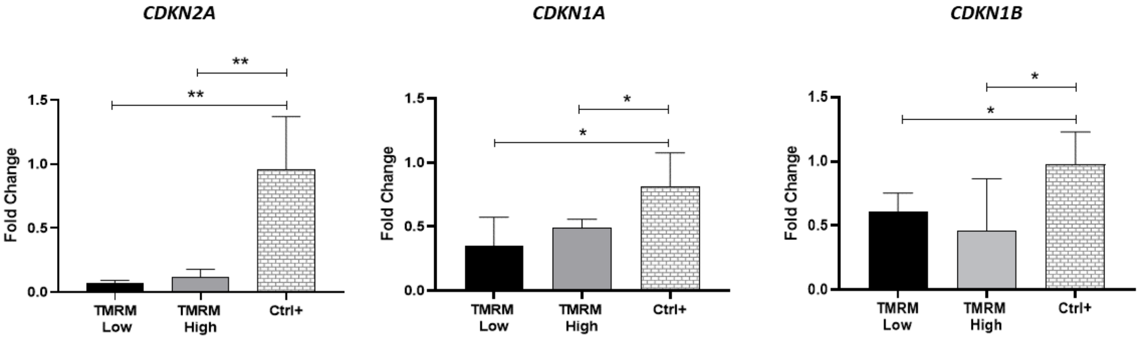

Supplementary Figure S2. Senescence gene expression in TMRM Low and High cells. Expression of genes involved in cell senescence: Cyclin-dependent kinase inhibitor 2A (*CDKN2A*, p16), Cyclin-dependent kinase inhibitor 1 (*CDKN1A*, p21) and Cyclin-dependent kinase inhibitor 1B (*CDKN1B*, p27). Data are represented as mean ± SD of the fold change. n=3 per group. Statistical differences were calculated significant as *$p < 0.05$, **$p < 0.01$ determined by Student t-test.

Supplementary Table S1. Real-time PCR primer sequences.

Mitochondrial DNA quantification			
**Gene Symbol**	**Gene Name**	**Forward Sequence (5′-3′)**	**Reverse Sequence (5′-3′)**
B2M	Beta-2-microglobulin	TTG TCT TTC AGC AAG GAC TGG	TGA TGC TGC TTA CAT GTC TCG
ND5	Mitochondrially Encoded NADH: Ubiquinone Oxidoreductase Core Subunit 5	GGG GAT TGT GCG GTG TGT G	CTT CTC CTA TTT ATG GGG GT
**Gene Expression analysis**			
ACAN	Aggrecan	ACC AGA CGG GCC TCC CAG AC	ACA GCA GCC ACA CCA GGA AC
ACTA2 (α-SMA)	Alpha smooth muscle actin	GCT CCC CTA AAT CCC AAG GC	ATC ACC TGA ATC CAG GAC GAT
ACTC1 (α-Sarcomeric Actin)	Alpha sarcomeric actin	TGT CCT GAG ACA CTC TTC	TGA TGC TAT TGT AAG TTG TT
B2M	β2-microglobulin	CAT TCC TGA AGC TGA CAC CAT TC	TGC TGG ATG ACG TGA GTA AAC C
COL2A1	Collagen Type II Alpha 1 Chain	TGA GGG CGC GGT AGA GAC CC	TGC ACA CAG CTG CCA GCC TC
COL10A1	Collagen Type X Alpha 1 Chain	GCC CAC AGG CAT AAA AGG CCC	GAA GGA CCT GGG TGC CCT CGA
CDKN2A (p16)	Cyclin-dependent kinase inhibitor 2A	CTT CCT GGA CAC GCT GGT	CAA ACC CAC AAA TGG TTT CC
CDKN1A (p21)	Cyclin-dependent kinase inhibitor 1	GGA AGA CCA TGT GGA CCT GT	GGA TTA GGG CTT CCT CTT GG
CDKN1B (p27)	Cyclin-dependent kinase inhibitor 1B	*ACC TGC AAC CGA CGA TTC TTC*	*GGG CGT CTG CTC CAC AGA*
COX4I2	Cytochrome c oxidase subunit 4I2	GGT GGA AGA CGA GGG ATG CA	CAG CTG GGT CCA GCT TCC CT
FABP4	Fatty acid binding protein 4	TTC ATA CTG GGC CAG GAA TTT	TCC ATC CCA TTT CTG CAC AT
FIS1	Mitochondrial fission 1 protein	GGA GGA ACA GCG GGA TTA CGT	CTT CAT GGC CTT GTC AAT GAG C
GATA-4	GATA Binding Protein 4	AGC CTG GCC TGT CAT CTC ACT	GGC CAG ACA TCG CAC TGA CT
KDR	Kinase Insert Domain Receptor	CCC TGC GAA GTA CCT TGG TT	TGG GGT GGG ACA TAC ACA AC
KLF4	Krüppel-like factor 4	ACA TTA ATG AGG CAG CCA CCT G	AGA CGC GAA CGT GGA GAA AG
MMP13	Matrix metallopeptidase 13	ATG CGG GGT TCC TGA TGT GG	GGC CCA GGA GGA AAA GCA TG
MDR-1	Multidrug-resistance	GGC TCC GAT ACA TGG TTT TCC	CCA GTG GTG TTT TTA GGG TCA TC

MFN2	Mitofusin 2	CCC CCT TGT CTT TAT GCT GAT GTT	TTT TGG GAG AGG TGT TGC TTA TTT C
MYC (c-Myc)	MYC	TTC TCT CCG TCC TCG GAT TCT CT	AGA AGG TGA TCC AGA CTC TGA CCT
NANOG1	Nanog Homeobox	AAT ACC TCA GCC TCC AGC AGA TG	TCG GTC ACA CCA TTG CTA TTC TTC
NKX2.5	NK2 Homeobox 5	AAG TGT GCG TCT GCC TTT	GTT GTC CGC CTC TGT CTT C
NOS3 (eNOS)	Endothelial Nitric Oxide Synthase	GTG GCT GGT ACA TGA GCA CT	TGG CTA GCT GGT AAC TGT GC
PPARGC1A *(*PGC1α*)*	Peroxisome proliferator-activated receptor gamma coactivator 1-alpha	ACT CAA GTG GTG CAG TGA CC	CTG GGT ACT GAG ACC ACT GC
PLIN1	Perilipin 1	CAT TGA GAA GGT GGT GGA GTA	CTT GGC CTT GGG AGA CTT
POU5F1 (Oct-4)	Octamer-binding transcription factor 4	TGG GCT CGA GAA GGA TGT G	TGT GCA TAG TCG CTG CTT GAT
PPARγ	Peroxisome proliferator-activated receptor gamma	ACA TAA AGT CCT TCC CGC TGA CCA	AAA CTG GCA GCC CTG AAA GAT GC
RUNX-2	Runt-related transcription factor 2	TCT GGC CTT CCA CTC TCA GT	GAC TGG CGG GGT GTA AGTA
SOD2	Superoxide dismutase 2	GCT GCA CCA CAG CAA GCA GTC C	CCA GCA ACT CCC CTT TGG GT
SOX9	SRY-Box Transcription Factor 9	GGA CCA GTA CCC GCA CTT GCA	GTT CTT CAC CGA CTT CCT CCG CCG
SPP1 *(Osteopontin)*	Secreted Phosphoprotein 1	GCC GAG GTG ATA GTG TGG TT	TGA GGT GAT GTC CTC GTC TG
TBX5	T-Box Transcription Factor 5	TAG CAG TGA CTT CCT ACC	ACG GGA TAT TCT TTA CTT T
TNNT2 (cTNT)	Cardiac muscle troponin T	CAC CTC AAG CAG GTG AAG AA	TCC ATT CCA CTC AGT GCA TC
